# Gender comparison of perceptual-cognitive learning in young athletes

**DOI:** 10.1038/s41598-024-59486-6

**Published:** 2024-04-15

**Authors:** Isabelle Legault, Jocelyn Faubert

**Affiliations:** 1 Collège Lionel-Groulx, Ste-Thérèse, QC Canada; 2 Faubert Lab, School of Optometry, Université de Montréal, Montreal, QC Canada

**Keywords:** Human behaviour, Cognitive neuroscience, Learning and memory

## Abstract

Elite athletes demonstrate higher perceptual cognitive abilities compared to non-athletes and those capacities can be trained. A recent study showed that differences were observed between male and female athletes in their cognitive abilities whereby male athletes showed superior perceptual abilities compared to female athletes. The purpose of this study was to investigate whether there were gender differences in athletes’ perceptual cognitive learning using a 3D-MOT tracking task. The study was performed on 72 young people from 16 to 22 years of age; athlete males and females and non-athlete males and females were distributed in four distinct groups. Five sessions comprised of three thresholds were performed with each participant. Results indicated that all participants benefited from training and significantly increased their speed thresholds. Initial scores showed that male athletes achieved higher speed thresholds than any other groups. Furthermore, after 5 weeks, female athletes obtained higher speed thresholds in comparison to their non-athlete counterparts. In conclusion, engaging in sporting activity is associated with improved perceptual-cognitive abilities and learning. The results support the notion that competitive sport-related activity is beneficial for perceptual-cognitive functions and emphasizes the benefits of participating in sport-related activities for improved brain function with an even greater impact for females.

## Introduction

To avoid collisions or optimize decision-making when interacting with the real world, it is essential to anticipate and predict the object positions that dynamically change in our environment. The human brain can easily process and track multiple dynamic objects in our visual field. When driving or walking in a crowd, those skills are essential. These skills are particularly developed in athletes who, through their sport, develop capabilities for actions that can be described as agile, flexible, and fast; they readily anticipate the position of their teammates and opponents, allowing them to make better decisions. They seem to have acquired expertise in the management of the information present in their environment, which is associated with skills that are often categorized as perceptual-cognitive abilities. To measure these abilities, it is possible to use a multiple object tracking (MOT) task, which consists of simultaneously tracking several moving objects among moving distractors. The MOT task requires controlled endogenous processes to select some objects (targets) and ignore others (distractors). These perceptual-cognitive abilities of the observer can be assessed by measuring the number of items tracked without error^1–3^ or by measuring the maximum speed at which tracking can be performed without error^4^. It has been repeatedly demonstrated that the moving object pursuit task is complex and involves attentional mechanisms requiring selective^5–7^ and sustained attention^8,9^. Selective attention refers to the ability to differentiate a relevant stimulus (target) from other stimuli (distractors) and to respond appropriately, depending on the task^10^. Sustained attention refers to the ability to maintain attention or an alert state over a varying period of time^11^. In addition, some authors have demonstrated the importance of 3-dimensional vision for performing multiple object pursuit tasks (3D-MOT)^4^. This creates a task that is more representative of reality, primarily by stimulating a larger portion of participants' visual field. The 3D-MOT task has been measured in several populations, including athletes, adolescents, children, and older adults, demonstrating different speed thresholds across groups^4,12–17^. A recent study confirmed the speed threshold advantage when using stereoscopic 3D-MOT over non-stereoscopic MOT reported in earlier studies^4,18^ and showed this advantage to be associated with an increased flow state as measured by heart rate variability in female soccer players when using 3D-MOT^19^.

In terms of perceptual-cognitive abilities, studies have observed gender differences across the lifespan^20–23^. Roudaia and Faubert^24^ showed that males had significantly higher attentional tracking speeds than females, indicating greater temporal resolution of attention. In addition, the researchers found slower reaction times in women and noted that these were negatively influenced by the presence of distractors, concluding that attentional functions are less efficient in women. Gender distinctions are also observed in athletes. A meta-analysis published by Voss et al.^25^ suggests that athletes perform better on tasks measuring cognitive and attentional abilities than female athletes, compared to the nonathlete control group. Supporting these results, a recent publication using the 3D-MOT in college students reported a distinction in speed thresholds between athlete and nonathlete males and females^26^. Specifically, male athletes demonstrated better perceptual-cognitive abilities than female athletes and control groups; they were able to perform the task at a higher speed.

Like physical development, perceptual-cognitive abilities can be improved in various populations; individuals experience an increase in their speed thresholds on the 3D-MOT task after training ^4,13,14,17,27–30^. More specifically, elite athletes obtained higher speed thresholds on a 3D-MOT task and can benefit, like amateurs and nonathletes, from the training. To explain these results in athletes, it is argued that physical training alters neural connections related to attentional processes, which would contribute to better performance on the 3D-MOT task^31^. Perceptual cognitive training can lead to better speed performance for both men and women^14,17,27,28^, but to our knowledge, no study has directly measured the effects of sports participation in men and women on perceptual-cognitive task training outcomes.

Although there have been many studies using the expert versus novice paradigm, there is still a lack of knowledge in how this differs as a function of gender and whether sports practice impacts on these differences. The fact that sports performance levels can have an impact on brain capacity as represented by learning functions for processing complex dynamic stimuli is relatively novel^13,32^ and given that there are known gender differences in processing complex dynamic stimuli^24^, this begs the question as to whether the participation in competitive sport activities can mitigate the observed gender difference.

The purpose of this experiment was to assess whether there were gender differences in athletes’ and nonathletes’ perceptual cognitive learning performance after training using a 3D-MOT tracking task. Based on the study by Legault Sutterlin-Guindon, Faubert^26^, we expected females to be less efficient than males, Furthermore, based on studies comparing elite athletes and amateurs, we should observe differences in absolute thresholds between male and female athletes, but the athletes' learning curves was anticipated to be similar.

## Results

The within-subject *Mauchly test of sphericity* was not significant; consequently, our data reached the criteria of sphericity. Furthermore, the between-subjects *test box* was not significant, revealing that the homogeneity in our data was respected. Additionally, our QQ plots reveal a normal distribution of our data.

A repeated-measures ANOVA revealed a main effect of Groups *F* (3,68) = 12.282, *p* < 0.01, η_p_^2^ = 0.351 and a main effect of session *F* (1, 4) = 2.481, *p* < 0.01. Furthermore, we did not obtain a significant session by group interaction, *F*(4,12) = 0,36, *p* = 0.494, which reveals that groups obtained similar learning functions. As shown in Table 1, post hoc comparison using Bonferroni correction showed differences between groups, and male athletes obtained better performance than the other groups (*p* < 0.0125 value to ascertain significance). Furthermore, female athletes obtained significant differences compared with nonathlete females.

**Table 1:** Bonferroni test.

Group (I)	Group (J)	Mean difference (I–J)	SD	*p value*
Multiple comparison				
AM	NAM	0.285*	0.0988	0.031*
	AF	0.431*	0.0988	< 0.001*
	NAF	0.573*	0.0988	< 0.0001*
AF	NAF	0.288*	0.0988	0.02*
	NAM	0.146	0.0988	0.868

A: Athlete/NA: Non-Athlete/M: Male/F: Female.

The error term is the mean square (Error) = 0.088.

*The mean difference is significant at the.05 level. Calculation based on observed means.

**Figure 1:**
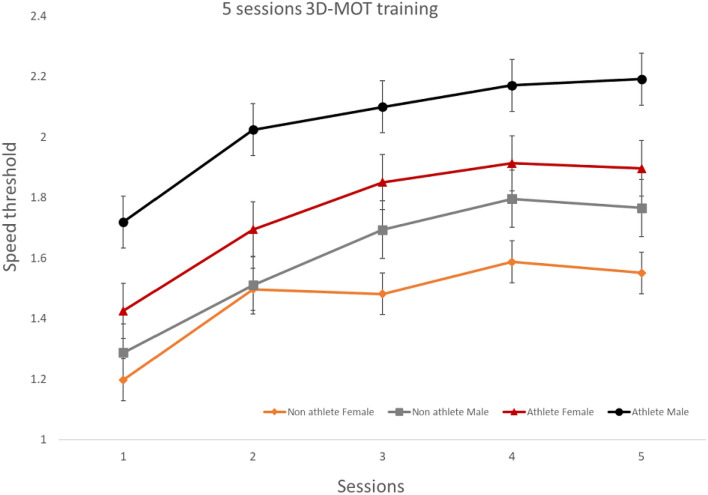
3D-MOT geometrical speed threshold means on a log scale.

First, as shown in Fig. 1, our results show a significant difference between sessions, meaning that participants improve after 5 sessions. These results are consistent with previous studies; after a 3D-MOT training protocol, participants improved from session to session^13,14,17,27–29^.

Second, our results showed a significant group effect. As observed in previous research, male athletes obtained higher speed thresholds than the other groups in the first session^26^. As expected, this difference was observed in our participants in the first session but also in subsequent sessions. Male athletes performed better than nonathletes and females during all 5 sessions. Furthermore, differences were observed between male and female athletes and nonathletes. Female athletes obtained higher speed thresholds than nonathlete females. However, their performances were similar to those of nonathlete males.

## Discussion

**Figure 2:**
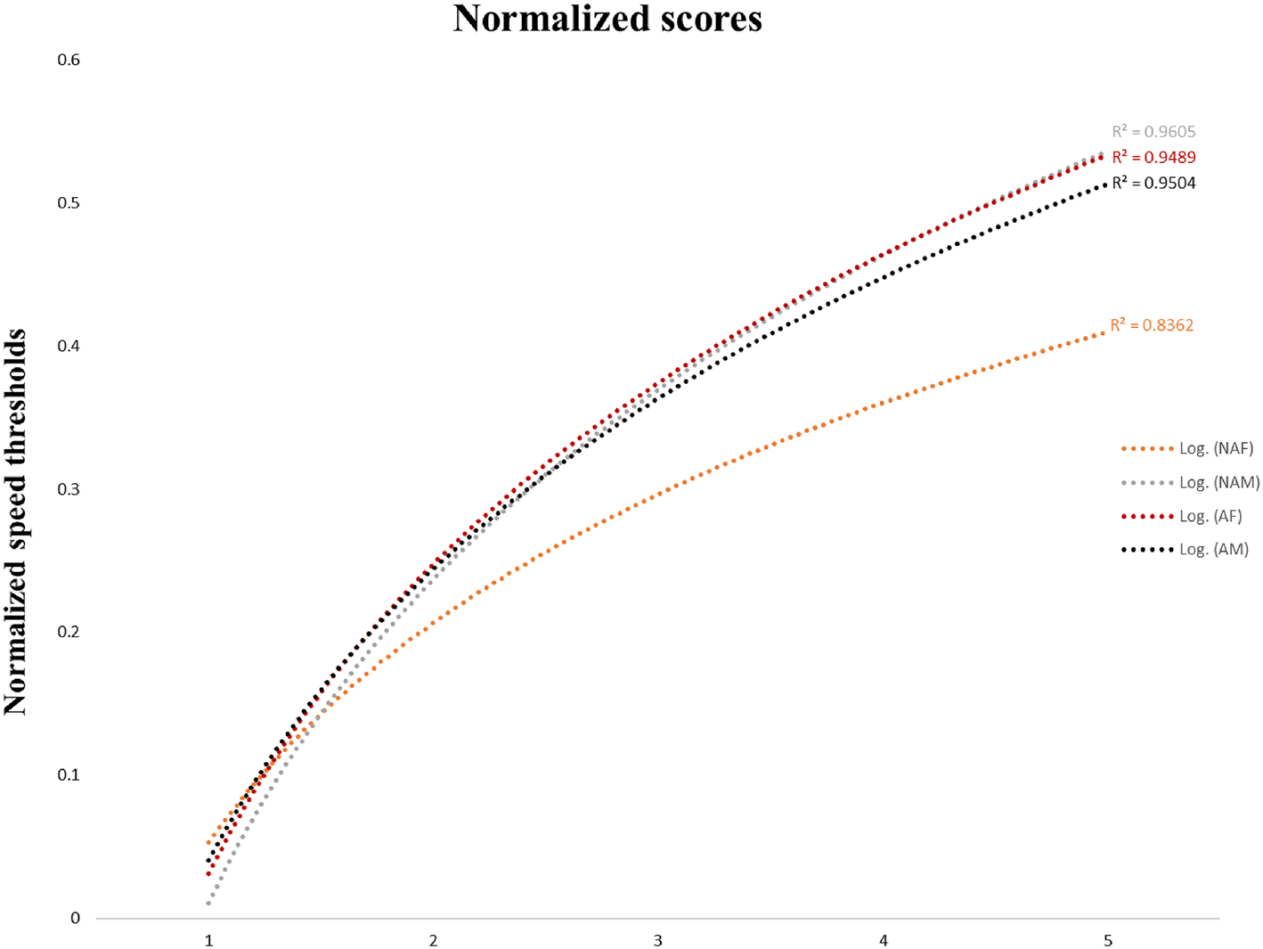
Represents the curve fit normalized functions obtained for each group after 5 sessions.

Figure 2 represents the curve fit normalized functions obtained for each group after 5 sessions. The dotted curve represents the regression curve (R2) on the normalized data (the threshold of the session—the threshold obtained in session 1). We can observe that the learning curves for male and female athletes are similar but differ significantly from the nonathlete female. The nonathlete females obtain a significantly less pronounced learning curve. This leads us to consider the influence of sports on cognitive abilities. As proposed by Roudaia and Faubert, differences remain between the male and female groups, where males obtained higher attentional tracking speed, which could explain the differences between males and females that we observed here^24^. However, female athletes obtained higher speed thresholds than their female counterparts, and these results indicate that sports have an impact on cognitive performance. Sport increases attentional abilities, regardless of gender.

Although male athletes achieve higher speed thresholds than nonathlete males, their learning curves are similar for these two groups (Fig. 2). In Faubert's 2013 article, differences are observable when elite-amateurs and nonathletes are compared to professional athletes^13^. Professional athletes obtain significantly steeper learning curves than elite-amateurs and nonathletes. We can assume that in our study, the level of athletic performance was not high enough to lead to differences between groups. It would be necessary to raise the level of these athletes to Tier 4: Elite/International Level or Tier 5: World Class^33^, to observe if differences emerge in their learning curves. Furthermore, the benefit of engagement in sports practice is greater for females than for males. This gain is easily observed with learning curves, where female athletes obtained similar learning curves to males, while nonathlete females obtained much less pronounced learning curves. Additionally, a distinction in learning curves for females appeared at an early stage in their sports engagement. It would be appropriate to encourage youth, especially females, to get involved in a sport, knowing that it is related to fundamental mental capacities for learning dynamic and complex situations and that sport can increase those capacities.

Furthermore, it has been shown that elite athletes demonstrate different brain neurophysiological characteristics. For instance, Wei et al.,^34^ showed that certain brain areas responsible for active motion integration of socially relevant information for interpreting body movements known as biological motion perception, are enhanced in elite athlete brains. Furthermore, researchers have observed structural differences between athletes and non-athletes^35–37^, as well as differences in hemodynamic response, with reduced energy consumption of the athlete brain^38^. It has also been demonstrated that elite athletes are superior at processing such dynamic cues in sports specific and non-sport specific situations^16^. Further, it was shown that training people on 3D-MOT results in improved biological motion capacities in some populations^14^, which implies that these neural networks share properties. It is therefore possible that such neural networks shown to be more prominent in athletes are also involved in a dynamic scene task such as 3D-MOT and could potentially explain why athletic groups show enhanced learning potentials for such tasks^13^.

Previous research has reproduced the results on different types of sports, so we can assume that male and female athletes with the same level of training and frequency in their sporting activities could achieve similar perceptual-cognitive learning benefits. Our study results imply that some differences reported in the literature between males and females in perceptual-cognitive capacities for processing complex dynamic stimuli can be mitigated by competitive sports participation. This implies that participating in sports activities does not only result in the well-known physical health benefits but may also transfer to benefits in fundamental perceptual-cognitive functions that are relevant for daily activities.

### Limitations

In this article, we did not look at the effect of player position on performance in the 3D-MOT task. This would have required a much larger number of participants and a control for each position. The current number of participants does not allow this analysis. In addition, we used participants from different team sports, so it might be interesting to compare results on the attentional task between different types of sport. We also assessed the effect of sport participation on attentional capacities, but we did not look at different levels of competition. It's possible that there were differences between the groups of male athletes, notably between those who played hockey, soccer, or football. Also, the level of competition is undoubtedly different between males and females, which may explain the difference between the two groups. There were also differences in the number of participants involved in each sport, and the sports played were different between male and female athletes. Although the result of the present study supports previous research involving a 3D-MOT task in athletes, the differences we observe in girls cannot be generalized outside collective sports and age group to which they belong. No previous research has compared the results obtained in female and male for a 3D-MOT task learning function, so it's difficult to generalize our findings to other populations. We would need to conduct research looking at individual sports in both female and male populations and compared them. Furthermore, there were no differences in learning curves between non-athlete males and female athletes. Both groups had similar results at the beginning and end of cognitive training. It is difficult to explain why female athletes are similar to non -athlete males for 3D-MOT learning functions. We cannot determine the upper limit of sports participation with females given the level of expertise (Tier 3) that is involve in our study. We can only presume that female elite athletes (Tier 4: Elite/International Level; or Tier 5: World Class)^33^ are more experienced and are exposed to more complex and faster field movement dynamics, would obtain higher performance that non-athlete males. Future research is needed before any conclusions can be drawn as to the extent of sports participation benefits to perceptual-cognitive functions in females.

## Conclusion

In conclusion, male athletes obtained higher speed thresholds than the other groups. Male athletes obtained similar learning curves to nonathlete male and females. It has been repeatedly demonstrated that practising sport is important for maintaining good physical health, but also essential for maintaining good mental and cognitive health^39–42^ The practice of sports, particularly through involvement in sports teams, appears to be particularly beneficial for improving cognitive capacity. Moreover, it seems even more important for young female athletes, who seem to significantly improve their perceptual-cognitive abilities compared to non athlete females. It's essential to encourage not only boys to take part in sports, but also girls, who seem to particularly benefit from sports for perceptual-cognitive learning transfer.

## Materials and methods

### Participants

Thirty-six athletes (18 boys: 17.9 years of age, σ 0.76 and 18 girls: μ: 18 years of age, σ 1.03) and thirty-six nonathletes (18 boys μ: 18.8 years of age, σ 1.29 and 18 girls μ: 18.2 years of ageσ 1.38) (range: 16–22 years of age) participated in this study. A power analysis was conducted using G*Power version 3.1.9.7^43^ for sample size estimation, the effect size is considered to be medium^44^. With a significance criterion of α = 0.05 and power = 0.80, the minimum sample size needed with this effect size is N = 48 for a repeated measured ANOVA. Thus, the obtained sample size of N = 72 is more than adequate to test the hypothesis. Participants were recruited from the college population, and they were, for the majority, in a preuniversity program. Ethical approval was obtained from the College Lionel Groulx Ethics Board (#2020-04), and written informed consent was obtained from all participants. The ethics board authorized the participants (aged 16 years old and over) to give their own consent based on the low risk factor in this study. All participants were naïve to the purpose of the experiment and had no previous experience with the 3D-MOT task. All subjects had normal or corrected-to-normal vision (6/6 or better) with normal stereoacuity as measured by the RANDOT Stereo Test (50 s of arc or better)^45^. Viewing was binocular. All participants completed the D2 test of attention, a screening measure for attention deficit disorder, and all scores were within the normal range. All participants were required to have had a history of fewer than two concussions and to be in excellent health at the time of testing. All athlete participants were members of a college sports team, as team sports generally solicit multifocal attention. Furthermore, it has been previously shown that athletes participating in different team sports demonstrated identical learning functions for the 3D-MOT task^13^. Consequently, it was hypothesized that the participants would demonstrate similar performance functions on this task. The athletes were involved in their sport during practice or matches at least twice a week, for at least 1 h per session. In the male athlete’s group, 5 were soccer players, 7 were football players and 6 were hockey players. In the female group, 5 were soccer players, 5 hockey players 5 flag football players and 3 were basketball players. Those athletes were involved in organised sports for a minimum of 5 years. As suggested by McKay and collaborators, the classification framework to describe our athletes is Tier 3 (Highly Trained/National Level)^33^. None of the participants in the non-athlete groups practiced any organized sport and had no previous experience in collective sport practice. Nonathlete participants were not involved in a regular sports practice (less than once a week), and they were not part of any sports teams within the last five years.

### Procedure

Participants were asked to wear an *HTC Oculus Vive* virtual reality system that allowed high-resolution 3D images. The task was briefly explained to the participant, there was no initial exposition to the task. The task began at a given speed of 1 in the software which represents 68 cm/s in virtual speed. The participants were seated and asked to stare at the fixation point, located straight ahead. Stimuli consisted of height spheres projected into a virtual cube. The anterior side of the cube measured 45° of visual angle.

### 3D-MOT paradigm

**Figure 3:**
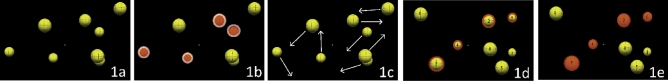
Five steps of the 3D-MOT task: (**a**) presentation phase, where 8 spheres are shown in a 3D volume space; (**b**) indexing phase, where 4 spheres (targets) change color (red) and are highlighted by a halo for 1 s; (**c**) movement phase, where the targets indexed in stage b return to their original form and color and all spheres move for 8 s crisscrossing and bouncing off each other and the virtual 3D volume cube walls that are not otherwise visible; and (**d**) identification phase, where the spheres come to a halt and the observer has to identify the 4 spheres originally indexed in phase (**b**). The spheres are individually tagged with a number so the observer can report the number. (**e**) Feedback was given to the observer.

The commercial version of the 3D-MOT speed threshold task, called NeuroTracker™ (CogniSens Inc.), was used to assess the perceptual cognitive abilities of the participants and the task was performed in accordance with the relevant guidelines and regulations**.** The task was projected in the Head Mounted Display(HMD). Five sessions comprising three blocks using the CORE program were performed with each participant. Each block consisted of 20 trials (total of 60 trials). At each session, the observers ran 3 consecutive COREs, lasting approximately 20 min. In average, each participant did two sessions per week, and there was a maximum of one week between session. The basic 3D-MOT trial sequence is presented in Fig. 3 and comprises four steps (see legend). The task began at a given speed, and at the end of the trial, if all four spheres were not correctly identified, the next trial decreased the speed. If the four spheres were correctly identified, then the next trial was faster. Twenty trials were presented to the participants, and the final speed threshold was calculated as the average of the last 4 reversals^26^. The experimental design was used in various research and has been demonstrated to be valid to measure perceptual-cognitive performance^4,13,14,16,19,27,28,46–48^.

### Data analysis

The task measured speed thresholds obtained by each participant after 3 blocks of 20 trials repeated on 5 sessions (total of 60 trials per subject). The speed value used for statistical analysis for the entire session is the average of the 3 block thresholds obtained for each of the participants. For each participant, we obtained five mean thresholds for the five experimental sessions. We compared, by a statistical analysis (repeated-measures ANOVA), whether differences remain between groups (main effect of groups) and between sessions (main effect of session) and evaluate whether significant interactions are obtained (session * groups). The effect size was determined using the Lambda square effect size. Furthermore, using a post hoc comparison, a Bonferroni correction was applied therefore we used 0.0125 value to ascertain significance.

## Data Availability

The data that support the findings of this study are available from the corresponding author upon reasonable request.

## References

[1] Cavanagh P, Alvarez GA (2005). Tracking multiple targets with multifocal attention. Trends Cogn. Sci..

[2] Pylyshyn ZW (1989). The role of location indexes in spatial perception: A sketch of the FINST spatial-index model. Cognition.

[3] Yantis S (1992). Multielement visual tracking: attention and perceptual organization. Cogn. Psychol..

[4] Faubert, J. & Sidebottom, L. The NeuroTracker System: Its role for perceptual-cognitive training of athletes and its potential impact on injury reductions and concussion management in sports. *Journal of clinical sports psychology* in press (2012).

[5] Flombaum JI, Scholl BJ, Pylyshyn ZW (2008). Attentional resources in visual tracking through occlusion: The high-beams effect. Cognition.

[6] Pylyshyn ZW (2006). Some puzzling findings in multiple object tracking (MOT): II. Inhibition of moving nontargets. Visual Cognit..

[7] Pylyshyn ZW, Haladjian HH, King CE, Reilly JE (2008). Selective nontarget inhibition in multiple object tracking. Visual Cognit..

[8] Doran MM, Hoffman JE (2010). The role of visual attention in multiple object tracking: Evidence from ERPs. Atten. Percept. Psychophys..

[9] Drew T, McCollough AW, Horowitz TS, Vogel EK (2009). Attentional enhancement during multiple-object tracking. Psychon. Bull. Rev..

[10] Armstrong C (1997). Selective versus sustained attention: A continuous performance test revisited. Clin. Neuropsychol..

[11] Cicerone KD (1997). Clinical sensitivity of four measures of attention to mild traumatic brain injury. Clin. Neuropsychol..

[12] Corbin-Berrigan LA, Kowalski K, Faubert J, Christie B, Gagnon I (2018). Three-dimensional multiple object tracking in the pediatric population: The NeuroTracker and its promising role in the management of mild traumatic brain injury. Neuroreport.

[13] Faubert J (2013). Professional athletes have extraordinary skills for rapidly learning complex and neutral dynamic visual scenes. Sci. Rep..

[14] Legault I, Allard R, Faubert J (2013). Healthy older observers show equivalent perceptual-cognitive training benefits to young adults for multiple object tracking. Front. Psychol..

[15] Legault I, Troje NF, Faubert J (2012). Healthy older observers cannot use biological-motion point-light information efficiently within 4 m of themselves. i-Perception.

[16] Romeas T, Faubert J (2015). Soccer athletes are superior to non-athletes at perceiving soccer-specific and non-sport specific human biological motion. Front. Psychol..

[17] Tullo D, Guy J, Faubert J, Bertone A (2018). Training with a three-dimensional multiple object-tracking (3D-MOT) paradigm improves attention in students with a neurodevelopmental condition: A randomized controlled trial. Dev. Sci..

[18] Plourde M, Corbeil M-E, Faubert J (2017). Effect of age and stereopsis on a multiple-object tracking task. PLoS ONE.

[19] Che X (2023). Two-dimensional and three-dimensional multiple object tracking learning performance in adolescent female soccer players: The role of flow experience reflected by heart rate variability. Physiol. Behavior..

[20] Feng J, Spence I, Pratt J (2007). Playing an action video game reduces gender differences in spatial cognition. Psychol. Sci..

[21] Geiser C, Lehmann W, Eid M (2008). A note on sex differences in mental rotation in different age groups. Intelligence.

[22] Jansen P, Kellner J, Rieder C (2013). The improvement of mental rotation performance in second graders after creative dance training. Creative Education.

[23] Voyer D, Voyer S, Bryden MP (1995). Magnitude of sex differences in spatial abilities: A meta-analysis and consideration of critical variables. Psychol. Bull..

[24] Roudaia E, Faubert J (2017). Different effects of aging and gender on the temporal resolution in attentional tracking. J. Vision.

[25] Voss MW, Kramer AF, Basak C, Prakash RS, Roberts B (2010). Are expert athletes ‘expert’ in the cognitive laboratory? A meta-analytic review of cognition and sport expertise. Appl. Cognitive Psych..

[26] Legault I, Sutterlin-Guindon D, Faubert J (2022). Perceptual cognitive abilities in young athletes: A gender comparison. PLOS ONE.

[27] Legault I, Faubert J (2012). Perceptual-cognitive training improves biological motion perception: Evidence for transferability of training in healthy aging. Neuroreport.

[28] Parsons B (2016). Enhancing cognitive function using perceptual-cognitive training. Clin. EEG Neurosci..

[29] Romeas T, Chaumillon R, Labbé D, Faubert J (2019). Combining 3D-MOT with sport decision-making for perceptual-cognitive training in virtual reality. Percept. Mot. Skills.

[30] Romeas T, Guldner A, Faubert J (2016). 3D-multiple object tracking training task improves passing decision-making accuracy in soccer players. Psychol. Sport Exerc..

[31] Nakata H, Yoshie M, Miura A, Kudo K (2010). Characteristics of the athletes' brain: Evidence from neurophysiology and neuroimaging. Brain Res. Rev..

[32] Mancı E, Günay E, Güdücü Ç, Herold F, Bediz CŞ (2023). The effect of the playing positions in basketball on measures of cognitive performance. J. Cognitive Enhanc..

[33] McKay AKA (2022). Defining training and performance caliber: A participant classification framework. Int. J. Sports Physiol. Perform..

[34] Wei G, Zhang Y, Jiang T, Luo J (2011). Increased cortical thickness in sports experts: A comparison of diving players with the controls. PLoS One.

[35] Di X (2012). Altered resting brain function and structure in professional badminton players. Brain Connect..

[36] Duru AD, Balcioglu TH (2018). Functional and structural plasticity of brain in elite karate athletes. J. Healthc. Eng..

[37] Hänggi J, Koeneke S, Bezzola L, Jäncke L (2010). Structural neuroplasticity in the sensorimotor network of professional female ballet dancers. Human Brain Mapp..

[38] Manci E, Deniz OC, Guducu C, Gunay E, Bediz CS (2021). Hemodynamic changes in athletes' brains: Is there any adaptation?. Gen. Physiol. Biophys..

[39] Hillman CH, Erickson KI, Kramer AF (2008). Be smart, exercise your heart: exercise effects on brain and cognition. Nat. Rev..

[40] Moreau D (2014). Software marketing: Can brain training boost cognition?. Nature.

[41] Moreau D, Conway AR (2014). The case for an ecological approach to cognitive training. Trends Cogn. Sci..

[42] Singh B (2023). Effectiveness of physical activity interventions for improving depression, anxiety and distress: An overview of systematic reviews. Br. J. Sports Med..

[43] Faul F, Erdfelder E, Lang AG, Buchner A (2007). G*Power 3: A flexible statistical power analysis program for the social, behavioral, and biomedical sciences. Behav. Res. Methods.

[44] Kang H (2021). Sample size determination and power analysis using the G*Power software. J. Educ. Eval. Health Prof..

[45] Leat S, St-Pierre J, Hassan-Abadi S, Faubert J (2001). The moving dynamic random dot stereosize test: Development, age norms, and comparison with the frisby, randot, and stereo smile tests. J. Pediatric Ophthalmol. strabismus.

[46] Chamoun M (2017). Cholinergic potentiation improves perceptual-cognitive training of healthy young adults in three dimensional multiple object tracking. Front. Human Neurosci..

[47] Michaels J (2017). Driving simulator scenarios and measures to faithfully evaluate risky driving behavior: A comparative study of different driver age groups. PLoS One.

[48] Roy, Y. & Faubert, J. *Significant changes in EEG neural oscillations during different phases of three-dimensional multiple object tracking task (3D-MOT) imply different roles for attention and working memory*. (2022).

